# Single‐cell profiling of peripheral blood reveals acute‐phase immune dysregulation in human scrub typhus

**DOI:** 10.1002/imt2.70167

**Published:** 2026-08-05

**Authors:** Yijia Guo, Yi Niu, Nihui Zhang, Xiao Han, Liyuan Zhang, Yongguo Du, Lifen Han, Xiuying Tian, Zhao Xu, Gaoyu Wang, Kwok‐Yung Yuen, Jasper Fuk‐Woo Chan, Dahua Xu, Feifei Yin

**Affiliations:** ^1^ Hainan Medical University‐The University of Hong Kong Joint Laboratory of Tropical Infectious Diseases, Key Laboratory of Tropical Translational Medicine of Ministry of Education, School of Basic Medicine Hainan Medical University Haikou China; ^2^ School of Intelligent Medicine and Technology (Big Data Research Center), Hainan Engineering Research Center for Health Big Data Hainan Medical University Haikou China; ^3^ Fujian Medical University Mengchao Hepatobiliary Hospital Fuzhou China; ^4^ Department of Infectious Disease the Second Affiliated Hospital of Hainan Medical University Haikou China; ^5^ State Key Laboratory of Emerging Infectious Diseases, Department of Microbiology, and Carol Yu Centre for Infection, Li Ka Shing Faculty of Medicine The University of Hong Kong Pokfulam Hong Kong Special Administrative Region China; ^6^ Department of Infectious Diseases and Microbiology The University of Hong Kong‐Shenzhen Hospital Shenzhen China

## Abstract

Integrated single‐cell RNA sequencing, spectral flow cytometry, immune‐receptor repertoire profiling, and plasma proteomics revealed coordinated peripheral immune remodeling during acute scrub typhus. Patients exhibited expansion of cytotoxic, clonally amplified CD8^+^ T cells with exhaustion‐associated features, depletion and apoptosis of CD4^+^ T cells, B‐cell contraction with enhanced plasmablast/plasma‐cell differentiation, and inflammatory myeloid remodeling. CD14^+^CXCR6^+^ and CD14^+^GNLY^+^ myeloid‐cell frequencies positively correlated with bacterial burden, defining prominent immune features of acute *Orientia tsutsugamushi* infection.

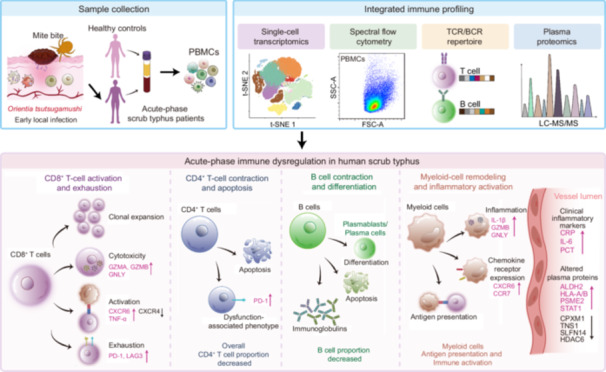

To the Editor,

Scrub typhus (ST) is a life‐threatening mite‐borne infection caused by *Orientia tsutsugamushi* (*O. tsutsugamushi*), an obligate intracellular bacterium transmitted by larval trombiculid mites [1, 2]. Although traditionally considered endemic to the Asia‐Pacific region, emerging reports indicate a broader geographic distribution and increasing global relevance [3]. Severe infection may progress to acute respiratory distress syndrome, hepatic injury, renal dysfunction, and multiorgan failure. Despite this substantial clinical burden, the immunopathological mechanisms underlying acute human scrub typhus remain poorly defined.

Protective immunity against intracellular pathogens generally requires coordinated innate and adaptive immune responses [4, 5]. Experimental studies of scrub typhus have implicated monocytes, dendritic cells, macrophages, and cytotoxic T lymphocytes in bacterial control, while also suggesting that excessive immune activation may drive tissue injury and disease severity [6, 7]. Transcriptional studies have further revealed inflammatory signatures and chemokine induction during infection; however, the cellular origins, differentiation states, and intercellular communication programs that shape these responses remain insufficiently characterized at single‐cell resolution [8, 9].

To address these gaps, we integrated single‐cell RNA sequencing (scRNA‐seq), spectral flow cytometry, immune‐receptor repertoire profiling, and plasma proteomics to define the peripheral immune landscape of acute scrub typhus. Our data identify cytotoxic T‐cell dysregulation and inflammatory myeloid remodeling as central features of acute disease.

## ACUTE SCRUB TYPHUS RESHAPES THE PERIPHERAL IMMUNE LANDSCAPE

We profiled peripheral blood mononuclear cells (PBMCs) from patients with acute scrub typhus (ST patients) and healthy controls (HCs) using scRNA‐seq, complemented by spectral flow cytometry and plasma proteomics. The discovery cohort comprised six patients with scrub typhus and three healthy controls for scRNA‐seq analysis. Independent validation included eight patients and eight controls for flow cytometry, and nine patients and six controls for plasma proteomics (Figure S1A–C). The reagents and gating strategies used for spectral flow cytometry were provided in Tables S1 and S2.

**FIGURE 1:**
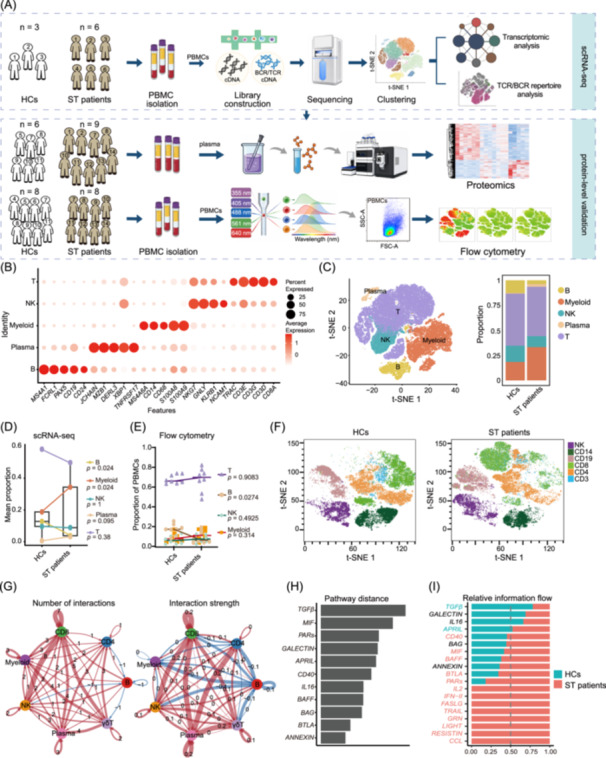
Study design and overview of peripheral immune remodeling in acute scrub typhus. (A) Schematic overview of the study design. Peripheral blood mononuclear cells (PBMCs) from patients with acute scrub typhus (ST patients; *n* = 6) and healthy controls (HCs; *n* = 3) were subjected to single‐cell RNA sequencing (scRNA‐seq) for transcriptional profiling and T cell receptor/B cell receptor (TCR/BCR) repertoire analysis. Key findings were further evaluated by spectral flow cytometry in an independent validation cohort (ST, *n* = 8; HCs, *n* = 8), and plasma proteomic profiling was performed to assess systemic protein alterations (ST patients, *n* = 9; HCs, *n* = 6). (B) Dot plot showing expression of canonical marker genes across the major immune populations. Dot size indicates the percentage of cells expressing each gene, and color indicates scaled average expression. (C) T‐distributed stochastic neighbor embedding (t‐SNE) visualization of the major PBMC populations identified by scRNA‐seq (left), and relative proportions of the five major immune populations in HCs and ST patients (right). (D) Unpaired dot plot shows sample‐level comparison of major immune‐cell proportions between HCs and ST patients by scRNA‐seq. Statistical significance was assessed using the two‐sided Mann–Whitney *U* test. (E) Unpaired dot plot shows flow cytometric comparison of the proportions of major immune populations between HCs and ST patients. Statistical significance was assessed using the two‐sided Mann–Whitney *U* test. (F) t‐SNE projection of flow‐cytometric data from HCs (left) and ST patients (right). (G) CellChat analysis comparing the number (left) and aggregate strength (right) of inferred ligand–receptor interactions across major immune cell populations between HCs and ST patients. (H) Pathway‐level analysis showing differences in the organization of predicted intercellular interaction networks between groups. (I) Relative information flow of major signaling pathways in HCs (green) and ST patients (red).

Among 14 patients, 10 had mild disease and four had severe disease, including two severe cases in the discovery cohort and two in the validation cohorts. All patients recovered following appropriate anti‐rickettsial treatment. Although participants were not individually matched, age and sex distributions did not differ significantly within assay cohorts (Table S3). Four circulating *O. tsutsugamushi* genotypes were identified, indicating that the study population was not restricted to a single bacterial lineage (Figure S1D). After quality control and batch correction, 61,366 cells were retained and annotated into major immune populations, including T cells, B cells, myeloid cells, NK cells, and plasma cells (Figure 1A–C and Table S4).

Comparative analysis revealed pronounced remodeling of the peripheral immune compartment during acute infection. Patients exhibited a significant contraction of B‐cell populations and relative expansion of myeloid cells, while total T‐cell proportions showed only a non‐significant decreasing trend (Figure 1D). Spectral flow cytometry independently confirmed contraction of the B‐cell compartment, while overall T‐cell abundance remained relatively stable (Figure 1E). Despite this stability at the total T‐cell level, t‐distributed stochastic neighbor embedding visualization showed marked redistribution of T‐cell subsets, indicating substantial compositional remodeling within the T‐cell compartment during acute infection (Figure 1F). Together, these findings indicate that acute scrub typhus induces selective remodeling of circulating immune populations rather than uniform systemic immune activation.

We performed transcriptome‐based ligand–receptor inference using CellChat. Patients showed an increased number of inferred interactions across several immune populations, whereas the aggregate predicted interaction strength was reduced in myeloid cells, B cells, and CD4^+^ T cells (Figure 1G). Pathway‐level inference indicated relative enrichment of MIF‐, BTLA‐, FASLG‐, and TRAIL‐associated interactions, together with reduced TGFβ‐ and APRIL‐associated interactions (Figures 1H,I, S2). These results should be interpreted as hypothesis‐generating predictions based on transcript abundance and curated ligand–receptor pairs, rather than evidence of biologically active intercellular signaling.

## T‐CELL PROFILING REVEALS DISRUPTED CD4^+^/CD8^+^ BALANCE AND A CYTOTOXIC, EXHAUSTION‐ASSOCIATED CD8^+^ T‐CELL STATE

**FIGURE 2:**
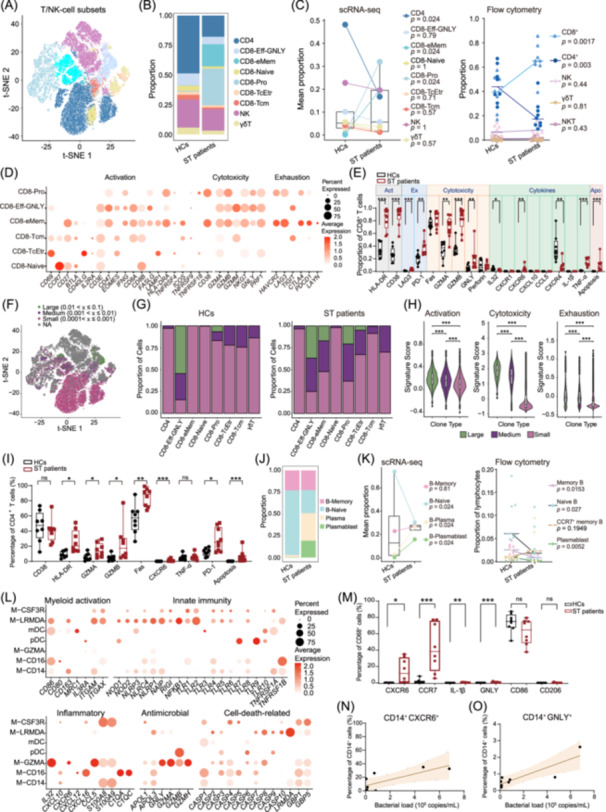
Acute scrub typhus induces coordinated remodeling of T‐cell, B‐cell, and myeloid‐cell compartments. (A) t‐SNE visualization of re‐clustered T/NK‐cell subsets, including CD4^+^ T cells, multiple CD8^+^ T‐cell subsets, NK cells, and γδ T cells. (B) Relative proportions of T/NK‐cell subsets in HCs and ST patients. (C) Unpaired dot plots show the frequencies of T/NK‐cell subsets determined by scRNA‐seq (left) and flow cytometry (right). Statistical significance was assessed using the two‐sided Mann–Whitney *U* test. (D) Dot plot showing expression of selected activation‐, cytotoxicity‐, and exhaustion‐associated genes across CD8^+^ T‐cell subsets. Dot size indicates the percentage of cells expressing each gene, and color indicates scaled average expression. (E) Flow cytometric analysis of activation (Act), exhaustion (Ex), cytotoxicity, cytokine‐related, and apoptosis‐associated markers (Apo) in CD8^+^ T cells. Statistical significance is indicated as *: adjusted *p* < 0.05, **: adjusted *p* < 0.01, and ***: adjusted *p* < 0.001. The raw *p* values obtained from two‐sided unpaired Mann–Whitney *U* tests were adjusted using the Benjamini–Hochberg procedure to control the false discovery rate. (F) t‐SNE visualization of T‐cell subsets colored according to TCR clone‐size categories. (G) Distribution of TCR clone sizes across T‐cell subsets in HCs and ST patients. (H) Comparison of activation, exhaustion, and cytotoxicity signature scores across clone‐size categories. Kruskal–Wallis test for overall comparison among the three groups (activation: *p* < 2.21 × 10^−16^; cytotoxicity: *p* < 2.21 × 10^−16^; exhaustion: *p* < 2.21 × 10^−16^), followed by pairwise comparisons using the Wilcoxon rank‐sum test. ***: *p* < 0.001. (I) Flow cytometric characterization of activation‐, cytotoxicity‐, and apoptosis‐associated markers in CD4^+^ T cells. Statistical significance is indicated as *: adjusted *p* < 0.05, **: adjusted *p* < 0.01, and ***: adjusted *p* < 0.001. The raw *p* values obtained from two‐sided unpaired Mann–Whitney *U* tests were adjusted using the Benjamini–Hochberg procedure to control the false discovery rate. (J) Relative proportions of B‐cell subsets in HCs and ST patients. (K) Unpaired dot plots show the frequencies of B‐cell subsets determined by scRNA‐seq (left) and flow cytometry (right). Statistical significance was assessed using the two‐sided Mann–Whitney *U* test. (L) Dot plot showing selected myeloid‐associated, innate immune, inflammatory, antimicrobial, and cell death‐related gene signatures across myeloid subsets. Dot size indicates the percentage of cells expressing each gene, and color indicates scaled average expression. (M) Flow cytometric analysis of inflammatory and migration‐associated markers in CD68^+^ myeloid cells from HCs and ST patients. Statistical significance was assessed using the two‐sided Mann–Whitney *U* test. Statistical significance is indicated as *: adjusted *p* < 0.05, **: adjusted *p* < 0.01, and ***: adjusted *p *< 0.001. (N, O) Correlation of bacterial load with the frequencies of CD14^+^CXCR6^+^ (N) and CD14^+^GNLY^+^ (O) myeloid cells. Associations between bacterial load and cell frequencies were assessed using simple linear regression. For CD14^+^CXCR6^+^ myeloid cells, *R* ^2^ = 0.5901, *p* = 0.0260, slope = 4.448 × 10^−5^, 95% CI = 7.450 × 10^−6^ to 8.151 × 10^−5^; for CD14^+^GNLY^+^ myeloid cells, *R* ^2^ = 0.7402, *p* = 0.0061, slope = 2.549 × 10^−6^, 95% CI = 1.040 × 10^−6^ to 4.057 × 10^−6^.

To define T‐cell dysregulation during acute scrub typhus, we performed high‐resolution re‐clustering of the T/NK‐cell subsets. This analysis identified multiple transcriptionally distinct CD8^+^ T‐cell subsets, including CD8‐Eff‐GNLY, CD8‐Naive, CD8‐TcEtr, CD8‐Tcm, CD8‐eMem, and CD8‐Pro, together with CD4^+^ T cells and γδ T cells (Figures 2A,B, S3A). Acute infection was associated with CD4^+^ T‐cell contraction and expansion of effector‐memory (CD8‐eMem) and proliferative CD8^+^ T‐cell (CD8‐Pro) subsets. Spectral flow cytometry further confirmed the expansion of total CD8^+^ T cells and the reduction of CD4^+^ T cells (Figure 2C).

Pseudotime analysis predicted progressive differentiation from naïve CD8^+^ T cells toward activated effector‐memory and proliferative states during acute infection (Figure S3B,C). Transcription factor analysis revealed distinct regulatory programs. In T cells, cytotoxic lymphocyte populations exhibited increased activity of NFIL3, BCL6B, and CREB5, consistent with enhanced cytotoxic differentiation and effector function (Figure S4A). CD8‐eMem and CD8‐Pro cells had the highest activation, cytotoxicity, and exhaustion scores. These subsets expressed high levels of cytotoxic molecules, including *GZMA*, *GZMB*, *PRF1*, and *GNLY*, together with activation‐associated markers such as *CD38* and *IFNG*. *HAVCR2*, *LAG3*, and *TIGIT* were markedly increased, indicating a hyperactivated, inflammation‐associated, and exhaustion‐prone CD8^+^ T‐cell state (Figure 2D). Functional enrichment network analysis revealed cytotoxic and inflammatory programs across T‐cell subsets. Pathways related to cytotoxicity, NK‐cell regulation, cytokine signaling, and chemotaxis were enriched mainly in CD8‐Eff‐GNLY, NK, and γδ T cells, whereas CD8‐Pro cells were characterized by cell‐cycle and proliferation‐associated pathways (Figure S4B).

Flow‐cytometric validation supported this phenotype. CD8^+^ T cells from patients displayed increased expression of HLA‐DR, CD38, LAG‐3, PD‐1, GZMA, GZMB, GNLY, CXCR6, and TNF‐α, accompanied by enhanced apoptosis (Figure 2E). These findings support expansion of a highly cytotoxic CD8^+^ T‐cell phenotype with exhaustion‐associated features, rather than a simple increase in conventional effector activation.

T‐cell receptor (TCR) repertoire analysis demonstrated oligoclonal expansion consistent with antigen‐driven selection in effector‐associated CD8^+^ T cell subsets (Figure 2F). Compared with healthy controls, patients showed reduced repertoire diversity and increased oligoclonality, with expanded clones enriched in activated and cytotoxic transcriptional programs. Large expanded clones had higher activation, cytotoxicity, and exhaustion scores than small clones, suggesting that expanded CD8^+^ T cells constitute a major cytotoxic population (Figure 2G,H).

In parallel, the CD4^+^ T‐cell compartment showed contraction and functional impairment. Although conventional activation markers were not substantially increased, CD4^+^ T cells exhibited elevated apoptosis, increased PD‐1 and Fas expression, and enrichment of apoptosis‐ and PI3K‐Akt‐related pathways (Figures 2I, S4C). Thus, acute scrub typhus involves exaggerated cytotoxic CD8^+^ T‐cell responses and disrupted helper T‐cell homeostasis. Whether these cells contribute primarily to bacterial control, inflammatory pathology, or both will require future tissue‐level and functional validation.

## ACUTE SCRUB TYPHUS IS ASSOCIATED WITH CONTRACTION AND TERMINAL DIFFERENTIATION OF THE B‐CELL COMPARTMENT

To define B‐cell alterations during acute scrub typhus, we re‐clustered the B‐cell compartment into four transcriptionally distinct subsets: B‐Naive, B‐Memory, Plasma, and Plasmablast (Figure S5A). Acute infection induced substantial B‐cell remodeling, characterized by depletion of naïve B cells and expansion of plasma‐cell and plasmablast populations, indicating a shift toward terminal differentiation (Figures 2J,K, S5B,C). Flow‐cytometric analysis further demonstrated reductions in naïve B cells and memory‐like B cells, together with increased apoptosis and Fas expression in circulating CD19^+^ B cells, suggesting impaired B‐cell homeostasis (Figures 2K, S5D).

B‐cell receptor (BCR) repertoire analysis revealed oligoclonal expansion predominantly within memory B‐cell, plasmablast, and plasma‐cell compartments, with significantly reduced overall BCR diversity (Figure S6A,B). Exploratory proteomics and transcriptional analyses further indicated enhanced antibody‐secreting differentiation, with increased immunoglobulin expression and enrichment of endoplasmic reticulum protein‐folding, secretory, humoral immune‐response, and classical complement pathways. Increased PRDM1 and IRF1 activity further supported plasmablast/plasma‐cell differentiation and antibody‐production programs during acute infection (Figure S6C–E).

Together, these findings indicate that acute scrub typhus is accompanied by simultaneous B‐cell depletion, apoptosis, and terminal differentiation, consistent with an antigen‐driven response and impaired humoral immune homeostasis [10].

## ACUTE SCRUB TYPHUS IS ASSOCIATED WITH INFLAMMATORY REMODELING OF THE MYELOID COMPARTMENT

To investigate innate immune remodeling during acute scrub typhus, we re‐clustered myeloid cells into seven transcriptionally distinct subsets, including CD14‐high monocytes (M‐CD14), CD16‐high monocytes (M‐CD16), CSF3R‐high cells (M‐CSF3R), GZMA‐high cells (M‐GZMA), LRMDA‐high cells (M‐LRMDA), myeloid dendritic cells (mDC), and plasmacytoid dendritic cells (pDCs) (Figure S7A,B). Acute infection was associated with pronounced remodeling of the myeloid compartment, characterized by a relative reduction of pDCs and selective enrichment of CSF3R‐high cells. Flow cytometry independently demonstrated increased proportions of CD16^+^ myeloid cells (Figure S7C). These data indicate that acute scrub typhus drives profound innate immune remodeling, marked by reduction of pDCs, emergency myelopoiesis, and a shift toward a pro‐inflammatory monocyte phenotype.

Functional profiling revealed marked transcriptional heterogeneity across myeloid subsets. M‐GZMA cells expressed elevated levels of granzymes (*GZMA*, *GZMB*, *GZMH*) and granulysin (*GNLY*), indicative of cytotoxic and direct antimicrobial effector functions. M‐CSF3R cells displayed a pronounced pro‐inflammatory transcriptional program, whereas M‐LRMDA cells showed signatures consistent with terminal differentiation and cell‐death‐related pathways, including upregulation of *LRMDA*, *GBP1*, and multiple caspases. Classical monocyte‐like populations (M‐CD14) showed upregulation of *TLR4*, *S100A8/A9*, and TNF‐associated inflammatory pathways, while dendritic‐cell subsets demonstrated remodeling of antigen presentation machinery and inflammatory‐signaling programs, with pDCs showing selective enrichment of endosomal pattern‐recognition receptor genes *TLR7* and *TLR9* (Figure 2L). Transcription‐factor analysis identified enrichment of inflammatory and activation‐associated regulators, including HMGB2, KLF4, and MAFB, alongside antigen‐presentation regulators such as SPIB, ZBTB46, and IRF4, consistent with inflammatory and immune‐activating remodeling (Figure S7D). Pseudotime analysis predicted differentiation paths originating from classical and non‐classical monocyte states and converging toward inflammatory and terminally differentiated phenotypes (Figure S8). Pathway analysis further identified innate immune activation, cytokine production, antigen presentation, complement activation, and humoral immunity programs (Figure S9A).

Spectral flow cytometry validated the inflammatory activation state of circulating myeloid cells. CD68^+^ myeloid cells from patients expressed higher levels of CXCR6, CCR7, GNLY, and IL‐1β, indicating enhanced inflammatory activation and expression of trafficking‐associated markers (Figure 2M). Notably, the proportions of CD14^+^CXCR6^+^ and CD14^+^GNLY^+^ cells positively correlated with bacterial burden, associating inflammatory myeloid activation with pathogen load in peripheral blood (Figure 2N,O). Exploratory plasma proteomics showed patterns broadly consistent with systemic inflammatory remodeling, although most individual protein differences did not remain significant after FDR correction (Figure S9B). Together, these findings indicate that acute scrub typhus is accompanied by extensive inflammatory remodeling of the myeloid compartment, which may contribute to both antimicrobial defense and systemic inflammation.

Overall, acute scrub typhus involved extensive innate and adaptive immune remodeling. This immune state was characterized by inflammatory myeloid‐cell remodeling, B‐cell contraction and terminal differentiation, and dysregulated intercellular communication networks. Expansion of cytotoxic CD8^+^ T cells was the dominant feature. Effector‐associated subsets, particularly CD8‐eMem and CD8‐Pro cells, exhibited enhanced cytotoxicity, activation, exhaustion‐related transcriptional programs, and oligoclonal TCR expansion. This phenotype resembles hyperactivated yet functionally dysregulated cytotoxic states observed in severe tuberculosis, acute brucellosis, and severe COVID‐19 [11, 12, 13].

In addition, the proportions of CD14^+^CXCR6^+^ and CD14^+^GNLY^+^ myeloid cells correlated positively with bacterial burden, suggesting that these markers may reflect infection‐associated myeloid activation. GNLY is best characterized as an antimicrobial effector of cytotoxic lymphocytes that contributes to the killing of intracellular bacteria and parasites, whereas CXCR6 promotes the tissue localization and persistence of activated lymphocytes in models of *Mycobacterium tuberculosis* and *Listeria monocytogenes* infection [14, 15, 16]. However, direct associations of either marker on CD14^+^ myeloid cells with pathogen load or disease severity have not, to our knowledge, been established in these infections. Thus, our findings identify CXCR6 and GNLY as candidate correlates, rather than validated biomarkers, of acute immune activation in scrub typhus. Larger longitudinal cohorts are needed to assess their relationships with disease severity, bacterial clearance, and treatment response.

Several limitations should be noted. First, the small discovery cohort and limited number of severe cases precluded robust severity‐stratified analyses, leaving it unclear whether the identified CD8^+^ T‐cell and myeloid phenotypes were consistent across disease severity categories. Second, the cross‐sectional, peripheral‐blood‐based design limited the assessment of longitudinal and tissue‐specific responses. Third, ligand–receptor interactions were computationally inferred rather than functionally validated. Larger longitudinal cohorts and mechanistic studies are needed to validate and extend these observations.

In summary, our study defines key features of peripheral immune alterations during acute *O. tsutsugamushi* infection. Acute scrub typhus was characterized by expansion of highly cytotoxic CD8^+^ T cells with exhaustion‐associated features, accompanied by inflammatory remodeling of the myeloid compartment. These findings provide a framework for understanding immune dysregulation in scrub typhus and may help prioritize candidate markers and immune pathways for future validation.

## AUTHOR CONTRIBUTIONS

**Yijia Guo**: Conceptualization; methodology; investigation; formal analysis; writing—original draft. **Yi Niu**: Conceptualization; investigation. **Nihui Zhang**: Investigation; formal analysis; software. **Xiao Han**: Formal analysis; writing—original draft. **Liyuan Zhang**: Investigation; resources. **Yongguo Du**: Investigation; resources. **Lifen Han**: Software. **Xiuying Tian**: Software; formal analysis. **Zhao Xu**: Formal analysis. **Gaoyu Wang**: Investigation; resources. **Kwok‐Yung Yuen**: Conceptualization; supervision. **Jasper Fuk‐Woo Chan**: Conceptualization; writing—review and editing. **Dahua Xu**: Conceptualization; writing—review and editing. **Feifei Yin**: Conceptualization; methodology; supervision; project administration; writing—original draft; writing—review and editing.

## CONFLICT OF INTEREST STATEMENT

The authors declare no conflicts of interest.

## ETHICS STATEMENT

The ethics application (No. HYLL‐2020‐061) was approved by the Research Ethics Committee of the Hainan Medical University. Written informed consent to participate in this study was obtained from all participants.

## Supporting information


**Figure S1:** Cohort characteristics and genotyping.
**Figure S2:** Altered intercellular communication networks during acute scrub typhus.
**Figure S3:** Transcriptional characterization and inferred transcriptional‐state trajectory analysis of CD8^+^ T cell subsets during acute scrub typhus.
**Figure S4:** Transcription factor and functional enrichment analyses of T‐cell subsets.
**Figure S5:** B‐cell subset annotation, apoptosis, and inferred transcriptional‐state trajectory during acute scrub typhus.
**Figure S6:** B‐cell receptor repertoire characteristics and immunoglobulin gene alterations during acute scrub typhus.
**Figure S7:** Myeloid‐cell annotation, subset composition, and transcription factor regulon activity during acute scrub typhus.
**Figure S8:** Inferred transcriptional‐state trajectory of myeloid‐cell subsets.
**Figure S9:** Functional enrichment of myeloid cells and plasma proteomic validation.

**Table S1:** Antibodies and staining reagents used in this study.
**Table S2:** Gating strategies used for spectral flow cytometry.
**Table S3:** Overall clinical cohort summary.
**Table S4:** Marker genes used for single‐cell RNA sequencing annotation.

## Data Availability

The data that support the findings of this study are openly available on GitHub at https://github.com/guoy8269-lgtm/ScRNA-Seq-Profiling-Acute-Phase-Immune-in-Human-Scrub-Typhus. Due to ethical and legal restrictions, de‐identified individual participant data and the corresponding data dictionary cannot be made publicly available. All data are available upon request to the corresponding author and subject to local rules and regulations. Supplementary materials (figures, methods, tables, graphical abstract, slides, videos, Chinese translated version, and updated materials) may be found in the online DOI or iMeta Science (http://www.imeta.science).
