# Supplementary material for: Single‐cell profiling of peripheral blood reveals acute‐phase immune dysregulation in human scrub typhus
Source: Imeta. 2026 Aug 5;5(4):e70167. doi: 10.1002/imt2.70167 (PMC13442654; doi:10.1002/imt2.70167)
Supplement: Supplementary file 1 [file IMT2-5-e70167-s002.docx]

**Supporting Information to**

# Single-cell profiling of peripheral blood reveals acute-phase immune dysregulation in human scrub typhus

## Running title: Acute-phase immune dysregulation in human scrub typhus

Yijia Guo^1#^, Yi Niu^1#^, Nihui Zhang^2#^, Xiao Han^3#^, Liyuan Zhang^4^, Yongguo Du^4^, Lifen Han^3^, Xiuying Tian^1^, Zhao Xu^1^, Gaoyu Wang^1^, Kwok-Yung Yuen^5,6^, Jasper Fuk-Woo Chan^5,6^*, Dahua Xu^2^*, Feifei Yin^1^*

^1^Hainan Medical University-The University of Hong Kong Joint Laboratory of Tropical Infectious Diseases, Key Laboratory of Tropical Translational Medicine of Ministry of Education, School of Basic Medicine, Hainan Medical University, Haikou, 571199, China

^2^School of Intelligent Medicine and Technology (Big Data Research Center), Hainan Engineering Research Center for Health Big Data, Hainan Medical University, Haikou, 571199, China

^3^Fujian Medical University Mengchao Hepatobiliary Hospital‌, Fuzhou, 350000, China

^4^Department of Infectious Disease, the Second Affiliated Hospital of Hainan Medical University, Haikou, 570311, China

^5^State Key Laboratory of Emerging Infectious Diseases, Department of Microbiology, and Carol Yu Centre for Infection, Li Ka Shing Faculty of Medicine, The University of Hong Kong, Pokfulam, Hong Kong Special Administrative Region, 999077, China

^6^Department of Infectious Diseases and Microbiology, the University of Hong Kong-Shenzhen Hospital, Shenzhen, 518000, China

^#^ These authors contributed equally: Yijia Guo, Yi Niu, Nihui Zhang, Xiao Han

*Correspondence: [yinfeifeiff@163.com (Feifei](mailto:yinfeifeiff@163.com%20(Feifei) Yin), [xudahua1209@muhn.edu.cn](mailto:xudahua1209@muhn.edu.cn) (Dahua Xu), and [jfwchan@hku.hk](mailto:jfwchan@hku.hk) (Jasper Fuk-Woo Chan)

**Supplementary methods**

**Study design**

This study was designed to characterize peripheral immune alterations during acute scrub typhus using an integrated multi-omics approach. PBMCs from patients with acute scrub typhus and healthy controls were profiled by scRNA-seq to define immune-cell composition, functional states, and transcriptional changes across major immune subsets. Key findings were further evaluated at the protein level in an independent spectral flow cytometry cohort, while systemic alterations in circulating proteins were assessed by plasma proteomics.

**Study participants and sample processing**

PBMCs and plasma were collected from patients enrolled through fever surveillance and epidemiological investigation of scrub typhus in Hainan, China, between 2021 and 2024. Patients presenting with acute undifferentiated febrile illness during the acute phase of infection (fever duration, 5–15 days) were screened, and scrub typhus was confirmed using a combination of nested PCR and serological testing. All included patients had no documented history of immunodeficiency and recovered after appropriate anti-rickettsial therapy.

For each participant, a total of 5 mL of EDTA-anticoagulated peripheral blood was collected for PBMC and plasma preparation. Healthy controls were recruited from residents of Hainan who had no fever within the preceding 72 h and tested negative for scrub typhus by PCR. Plasma was separated by centrifugation at 350 g for 10 min, and PBMCs were isolated from the remaining blood by Ficoll-Paque density-gradient centrifugation before cryopreservation. For scRNA-seq, samples were additionally screened by next-generation sequencing to exclude major bacterial or viral co-infections.

For scRNA-seq, PBMCs from 6 patients with acute scrub typhus and 3 healthy controls were analyzed. For flow cytometric validation, an independent cohort comprising 8 patients and 8 healthy controls was used. Plasma proteomic profiling was performed in 9 patients and 6 healthy controls. Laboratory abnormalities of the enrolled patients are summarized in Table S3. Disease severity was defined according to previously published clinical criteria, and peripheral leukocyte proportions were summarized in a broader clinical cohort of 14 patients; sample information for the scRNA-seq, flow cytometry, and plasma proteomics cohorts is provided in Table S3 [1]. The overall distributions of major immune populations were broadly concordant between scRNA-seq and spectral flow cytometry, supporting the reproducibility of the observed immune-cell composition changes (Figure S1B). Age distributions were compared between groups using the Mann–Whitney U test, as appropriate. Sex distributions were compared using Fisher’s exact test. Statistical analysis revealed no significant difference between the groups (Figure S1C and Table S3).

**10× Genomics single-cell library preparation and sequencing**

Single-cell libraries were generated using the Chromium Next GEM Single Cell 5’ Reagent Kit v2.0 (10× Genomics, Pleasanton, CA, USA) according to the manufacturer’s instructions. Briefly, cell suspensions were loaded onto a Chromium Controller to generate single-cell gel bead-in-emulsions (GEMs). Following reverse transcription and cDNA amplification, sequencing libraries were prepared by end repair, A-tailing, adaptor ligation, and PCR amplification. Final 5’ libraries were sequenced on a NovaSeq X Plus Sequencing System (Illumina, San Diego, CA, USA).

**scRNA-seq preprocessing and transcriptomic analysis**

Raw sequencing data were processed using Cell Ranger v3.1.0 (10× Genomics) for demultiplexing, alignment, and UMI counting. Cells with mitochondrial transcript content ≥ 20%, fewer than 250 detected genes, or fewer than 500 UMIs were excluded. Doublets were identified and removed using DoubletFinder v2.0.3. Downstream analyses were performed in Seurat v5.3.1 [2]. Count matrices were normalized using LogNormalize, variable genes were identified using the vst method, and scaled expression matrices were generated for downstream analysis. Batch effects were corrected using Harmony based on the first 20 principal components. Clustering was performed on a shared nearest-neighbor graph using the Louvain algorithm (resolution = 0.2) [3], and dimensionality reduction was visualized by t-SNE using the first 10 principal components. Cell identities were assigned using SingleR v2.4.1 with the Monaco Immune reference and were further refined based on canonical marker genes [4]. PBMCs were resolved into 20 immune subpopulations, including 8 T-cell subsets (CD4, CD8-Eff-GNLY, CD8-eMem, CD8-Naïve, CD8-Pro, CD8-TcEtr, CD8-Tcm, γδT), 7 myeloid-cell subsets (M-CD14, M-CD16, M-CSF3R, M-GZMA, M-LRMDA, M-mDC, M-pDC), 4 B-cell subsets (B Memory, B Naïve, B Plasma, B Plasmablast), and NK cells. Subset reclustering analyses for lymphoid, myeloid, and B-cell compartments were performed using the same preprocessing framework. Marker genes are provided in Table S4.

**Differential expression and pathway enrichment analysis**

Differentially expressed genes (DEGs) between patients and controls were identified using Seurat’s FindMarkers function with the Wilcoxon rank-sum test and Benjamini–Hochberg correction for multiple testing [5]. Gene set enrichment analysis (GSEA) was performed using clusterProfiler on ranked log_2_ fold-change lists for each immune subset. Both Gene Ontology Biological Process and KEGG pathway gene sets were tested, with minGSSize = 10, maxGSSize = 500, and a false discovery rate (FDR) threshold of < 0.05 for significance. A complete list of DEGs (FDR < 0.05 and |log_2_FC| > 0.25) is provided in GitHub repository: https://github.com/guoy8269-lgtm/ScRNA-Seq-Profiling-Acute-Phase-Immune-in-Human-Scrub-Typhus [6,7].

**Transcription-factor regulation, developmental trajectory analysis and cell communication analysis**

Transcription factor (TF) regulatory activity was inferred using pySCENIC (v0.12.1) for each subcluster identified from the scRNA-seq data. Motif-ranking databases required for RcisTarget were downloaded from the cisTarget database [8]. GENIE3 was used to infer co-expression-based TF-target modules within each cell type. Candidate regulons were then refined by motif enrichment analysis, and regulon activity in individual cells was quantified using AUCell to estimate TF activity.

Monocle2 (v2.30.1) was used to infer pseudotime ordering of major immune-cell populations. Differentially expressed genes among cell clusters were selected as ordering genes for DDRTree-based dimensionality reduction and cell ordering. Pseudotime values and inferred transcriptional trajectories were generated using the default parameters of Monocle2 [9]. Pseudotime analysis provides a computational ordering of transcriptional states and does not establish longitudinal cell-state transitions.

Potential intercellular communication among immune-cell populations was inferred using CellChat (v1.6.1) based on curated ligand–receptor interactions [10]. Cells from all participants within the same group were merged into a single group-level CellChat object, and inferred communication networks were compared between patients with acute scrub typhus and healthy controls. Communication probabilities and the statistical significance of ligand–receptor interactions were estimated using the default one-sided permutation test with 100 permutations. Cell populations containing fewer than 10 cells were excluded from the analysis, and interactions with permutation-test *p* values < 0.05 were considered statistically significant.

Differences in inferred interactions between groups were visualized using CellChat functions, including netVisual_bubble, based on the estimated communication probabilities, probability differences, and unadjusted permutation-test *p* values. Accordingly, these analyses were considered exploratory and hypothesis-generating, and the inferred interactions should not be interpreted as direct evidence of biologically active intercellular signaling.

**T-cell receptor and B-cell receptor repertoire analysis**

Single-cell 5’ V(D)J sequencing was performed and sequenced using the same workflow described above. Gene-expression and V(D)J libraries were generated from the same 5’ GEM emulsions, enabling cell-barcode-level integration of clonotype and transcriptomic information. Clonotype information was imported into scRepertoire and integrated with the single-cell transcriptome object. Clones were classified into five size categories (Single, Small, Medium, Large, and Hyperexpanded) based on CDR3 abundance, and their absolute numbers and proportions were visualized across cell subsets, patient groups, and disease states.

Activation, cytotoxicity, and exhaustion signature scores were calculated for individual cells using the AddModuleScore function in Seurat. Individual cells were used for visualization but were not treated as independent biological replicates. The cytotoxicity and exhaustion signatures each contained six genes and were derived from the gene sets reported by Jia et al. [11]. The activation signature contained 25 genes and was derived from the gene set reported by Xu et al. [12]. These signature scores were integrated with clonotype information to evaluate functional transcriptional states across clonotype-size categories. For statistical comparisons among the Small (0.0001 < $\text{x}$ ≤ 0.001), Medium (0.001 < $\text{x}$ ≤ 0.01), and Large (0.01 < $\text{x}$ ≤ 0.1) clonotype-size categories (Figure 2H), scores were aggregated by participant and clonotype-size category, and the mean score for each participant-category combination was used as the statistical observation. Overall differences among the three clonotype-size categories were assessed using the Kruskal–Wallis test, followed by post hoc pairwise comparisons using the Wilcoxon rank-sum test.

**Flow cytometric immunophenotyping**

Cryopreserved PBMCs (1 × 10^5^ to 6 × 10^5^ cells) were thawed and cultured in RPMI-1640 (Gibco, Thermo Fisher Scientific, Waltham, MA, USA) supplemented with 10% serum (Gibco, Thermo Fisher Scientific, Waltham, MA, USA) at 37°C for flow cytometric phenotyping. Cells were stained with Zombie Aqua for viability discrimination and then incubated with fluorochrome-conjugated surface antibodies. Data were acquired on a 5-laser Cytek Aurora instrument (Cytek Biosciences, Fremont, CA, USA). Antibody panels are listed in Supplementary Table S1. Spectral flow cytometry data were analyzed in FlowJo v10.8.1, and marker co-expression within predefined immune subsets was quantified in GraphPad Prism v10.4.2 using the unpaired Mann–Whitney U test. Panel-specific gating strategies are listed in Table S2.

**Flow cytometric cytokine and intracellular marker analysis**

For intracellular cytokine and functional-marker analysis, PBMC samples prepared as described above were processed using the corresponding antibody panels listed in Table S1 and S2. Raw FCS files were quality-controlled using FlowAI and downsampled prior to dimensionality-reduction analysis. t-SNE visualization and downstream quantification were performed after standard preprocessing. Statistical comparisons of cell proportions and marker expression were conducted in GraphPad Prism v10.4.2 using the unpaired Mann–Whitney U test. For Figure 2E, I, M, S5D, statistical significance is indicated as *: adjusted *p* < 0.05, **: adjusted *p* < 0.01, and ***: adjusted *p* < 0.001. The raw *p* values obtained from two-sided unpaired Mann–Whitney U tests were adjusted using the Benjamini–Hochberg procedure to control the false discovery rate.

**Quantification of *O. tsutsugamushi* load and genotyping**

To quantify bacterial burden, total genomic DNA was extracted from 200 μL of whole blood using the QIAamp DNA Mini Kit (Qiagen, Germany). Quantitative PCR targeting the *tsa56* gene was performed using TB Green Premix Ex Taq II (Takara, Japan) on a CFX96 Real-Time PCR System. The primer sequences were 5’–GGCCAAGTTAAACTCTATGCTGA–3’ (forward) and 5’–CATTAATTGCTACACCAAGTGC–3’ (reverse), generating an amplicon of approximately 163 bp. Cycling conditions were 95°C for 30 s, followed by 40 cycles of 95°C for 5 s and 60°C for 30 s, with melt-curve analysis performed at the end of amplification. Bacterial copy number was calculated using a plasmid standard curve ranging from 10^1^ to 10^8^ copies per reaction.

For genotyping, nested PCR targeting *tsa56* was performed as previously described, generating a 483-bp amplicon. Positive amplicons were subjected to bidirectional Sanger sequencing on an ABI 3730xl DNA Analyzer (Sangon Biotech, Shanghai, China). Sequence alignment was performed using ClustalW in MEGA-X v11.0.11. Pairwise sequence identities were calculated using MegAlign (DNASTAR), and phylogenetic trees were inferred using the maximum-likelihood method with the best-fit nucleotide substitution model implemented in MEGA-X. Node support was assessed using 1,000 bootstrap replicates (Figure S1D).

**Plasma proteomic analysis**

Hemolyzed samples were excluded from proteomic analysis based on visual inspection. Proteomic analysis was performed using plasma from 9 patients with scrub typhus and 6 healthy controls. Samples were thawed on ice and subjected to protein extraction using magnetic beads. For each sample, 500 ng of total peptides was analyzed using nano-UPLC (Vanquish Neo) coupled to an Orbitrap Astral mass spectrometer (Thermo Fisher Scientific, Waltham, MA, USA) equipped with a nano-electrospray ion source. Peptides were separated on an EASY-Spray HPLC column (150 μm × 15 cm; Thermo Fisher Scientific). Mobile phase A consisted of H₂O with 0.1% formic acid, and mobile phase B consisted of 80% acetonitrile with 0.1% formic acid. Data-independent acquisition (DIA) was performed in positive-ion mode. MS1 scans were acquired at 240,000 resolution over m/z 380–980, and MS2 scans were acquired over m/z 150–2000 with an AGC target of 5 × 10^4^, maximum injection time of 3 ms, HCD normalized collision energy of 25%, and an isolation window of 2 m/z.

Raw files were processed in Spectronaut v19.0.240604.62635 using the built-in Pulsar search engine and searched against the reviewed human UniProt database. Up to two missed cleavages were allowed. The false discovery rate was set to 0.01 at both the peptide-spectrum match and peptide levels. Initial precursor and fragment mass tolerances were set to 20 ppm. Differentially abundant proteins were first screened using *p* < 0.05 and fold change ≤ 0.83 or ≥ 1.20, and then subjected to multiple-testing correction using the fdrtool package in R. Proteins that remained significant after multiple-testing correction were considered robustly altered and were used for the primary interpretation of the proteomic data. The complete differential-abundance table, including both adjusted and unadjusted statistical results, has been deposited in the GitHub repository: <https://github.com/guoy8269-lgtm/ScRNA-Seq-Profiling-Acute-Phase-Immune-in-Human-Scrub-Typhus>. FDR-adjusted *q* values were calculated across all quantified proteins passing quality control. Proteins with *q* < 0.05 (n = 17) were considered statistically significant after multiple-testing correction. Proteins with nominal *p* < 0.05 but *q* ≥ 0.05 (n = 937) are displayed as exploratory candidates only and were not used as confirmatory evidence.

**REFERENCES**

1. Sriwongpan, Pamornsri, Pornsuda Krittigamas, Pacharee Kantipong, Naowarat Kunyanone, Jayanton Patumanond, Sirianong Namwongprom. 2013. “Clinical indicators for severe prognosis of scrub typhus.” *Risk Management and Healthcare Policy* 6: 43–49. <https://doi.org/10.2147/RMHP.S52470>
2. Butler, Andrew, Paul Hoffman, Peter Smibert, Efthymia Papalexi, Rahul Satija. 2018. “Integrating single-cell transcriptomic data across different conditions, technologies, and species.” *Nature Biotechnology* 36: 411–420. <https://doi.org/10.1038/nbt.4096>
3. Bhattacharya, Namrata, Swagatam Chakraborti, Stuti Kumari, Bernadette Mathew, Abhishek Halder, Sakshi Gujral, Krishan Gupta, *et al*. 2025. “Network based simultaneous embedding of cells and marker genes from scRNA-seq studies.” *Briefings in Bioinformatics* 26: bbaf537. <https://doi.org/10.1093/bib/bbaf537>
4. Aran, Dvir, Agnieszka P. Looney, Leqian Liu, Esther Wu, Valerie Fong, Austin Hsu, Suzanna Chak, *et al*. 2019. “Reference-based analysis of lung single-cell sequencing reveals a transitional profibrotic macrophage.” *Nature Immunology* 20: 163–172. <https://doi.org/10.1038/s41590-018-0276-y>
5. Camp, J. Gray, Keisuke Sekine, Tobias Gerber, Henry Loeffler-Wirth, Hans Binder, Malgorzata Gac, Sabina Kanton, *et al*. 2017. “Multilineage communication regulates human liver bud development from pluripotency.” *Nature* 546: 533–538. <https://doi.org/10.1038/nature22796>
6. Ashburner, Michael, Catherine A. Ball, Judith A. Blake, David Botstein, Heather Butler, J. Michael Cherry, Allan P. Davis, *et al*. 2000. “Gene ontology: tool for the unification of biology.” *Nature Genetics* 25: 25–29. <https://doi.org/10.1038/75556>
7. Kanehisa, Minoru, Susumu Goto. 2000. “KEGG: kyoto encyclopedia of genes and genomes.” *Nucleic Acids Research* 28: 27–30. <https://doi.org/10.1093/nar/28.1.27>
8. Aibar, Sara, Carmen Bravo González-Blas, Thomas Moerman, Vân Anh Huynh-Thu, Hana Imrichova, Gert Hulselmans, Florian Rambow, *et al*. 2017. “SCENIC: single-cell regulatory network inference and clustering.” *Nature Methods* 14: 1083–1086. <https://doi.org/10.1038/nmeth.4463>
9. Qiu, Xiaojie, Qi Mao, Ying Tang, Li Wang, Raghav Chawla, Hannah A Pliner, Cole Trapnell. 2017. “Reversed graph embedding resolves complex single-cell trajectories.” *Nature Methods* 14: 979–982. <https://doi.org/10.1038/nmeth.4402>
10. Jin, Suoqin, Christian F. Guerrero-Juarez, Lihua Zhang, Ivan Chang, Raul Ramos, Chen-Hsiang Kuan, Peggy Myung, *et al*. 2021. “Inference and analysis of cell-cell communication using CellChat.” *Nature Communications* 12:1088. <https://doi.org/10.1038/s41467-021-21246-9>
11. Jia, Yongxu, Baifeng Zhang, Chunyang Zhang, Dora Lai-Wan Kwong, Zhiwei Chang, Shanshan Li, Zehua Wang, *et al*. 2023. “Single-cell transcriptomic analysis of primary and metastatic tumor ecosystems in esophageal squamous cell carcinoma.” *Advanced Science* 10: e2204565. <https://doi.org/10.1002/advs.202204565>
12. Xu, Dahua, Nihui Zhang, Yutong Shen, Dehua Zheng, Zhizhou Xu, Peihu Li, Jiale Cai, *et al*. 2024. “Single-cell sequencing analysis reveals the dynamic tumour ecosystems of primary and metastatic lymph nodes in nasopharyngeal carcinoma.” *Journal of Cellular and Molecular Medicine* 28: e70137. <https://doi.org/10.1111/jcmm.70137>

**Supplementary figures**

**Figure S1 Cohort characteristics and genotyping.** (A) Peripheral leukocyte proportions in 14 ST patients. (B) Immune-cell proportions measured by flow cytometry (F) and scRNA-seq (S). (C) Age and sex distributions among the cohorts used for scRNA-seq, flow cytometry, and plasma proteomic analyses. (D) Phylogenetic analysis of the *tsa56* gene from clinical isolates, showing that the enrolled patients were infected with multiple circulating *O. tsutsugamushi* genotypes representative of the major endemic lineages in Hainan Province.

**Figure S2 Altered intercellular communication networks during acute scrub typhus.** (A) Marker genes employed for T-cell and NK-cell classification. (B) Bubble plots showing ligand–receptor interactions with significantly increased (top) or decreased (bottom) inferred communication probabilities in patients with acute scrub typhus compared with healthy controls.

**Figure S3 Transcriptional characterization and inferred transcriptional-state trajectory analysis of CD8⁺ T cell subsets during acute scrub typhus.** (A) Dot plot showing representative marker genes used for T-cell and NK-cell subset annotation. (B) Pseudotime trajectory analysis of six CD8^+^ T cell subsets, colored by subset identity, pseudotime, and disease group. (C) Heatmap showing pseudotime-dependent gene-expression dynamics, with representative enriched biological processes shown for each gene module.

**Figure S4 Transcription factor and functional enrichment analyses of T-cell subsets.** (A) Heatmap showing differential transcription factor regulon activity across T-cell subsets from healthy controls (HCs) and ST patients. (B) Functional enrichment network analysis showing immune-related pathways enriched across T-cell subsets. Node color indicates cell-type identity and node size reflects the number of genes contributing to each pathway. (C) Gene set enrichment analysis of cell death-related pathways in CD4^+^ T cells and CD8-eMem cells from patients compared with healthy controls.

**Figure S5 B-cell subset annotation, apoptosis, and inferred transcriptional-state trajectory during acute scrub typhus.** (A) t-SNE visualization and marker genes used for B-cell classification. (B) Heatmap showing pseudotime-dependent gene-expression dynamics, with representative enriched biological processes shown for each gene module. (C) Pseudotime trajectory analysis of four B-cell subsets, colored by subset identity, pseudotime, and disease group. (D) Flow-cytometric quantification of apoptotic CD19^+^ B cells and FAS-expressing CD19^+^ B cells in healthy controls (HCs) and ST patients. Statistical significance is indicated as *: adjusted *p* < 0.05, and ***: adjusted *p* < 0.001. The raw *p* values obtained from two-sided unpaired Mann–Whitney U tests were adjusted using the Benjamini–Hochberg procedure to control the false discovery rate.

**Figure S6 B-cell receptor repertoire characteristics and immunoglobulin gene alterations during acute scrub typhus.** (A) t-SNE visualization of B-cell subsets colored according to BCR clone-size categories and distribution of clone sizes across B-cell subsets in healthy controls (HCs) and ST patients. (B) Comparison of BCR diversity indices, including Shannon, inverse Simpson, Chao, ACE, and inverse Pielou indices. (C) Volcano plot showing differentially expressed immunoglobulin genes between patients and healthy controls. (D) Dot plot showing differential expression of immunoglobulin variable-region genes across B-cell subsets. (E) Differential transcription factor activity across B-cell subsets in healthy controls (HCs) and ST patients.

**Figure S7 Myeloid-cell annotation, subset composition, and transcription factor regulon activity during acute scrub typhus.** (A) t-SNE visualization of myeloid-cell subsets and expression of representative marker genes. (B) Dot plot of marker genes used for myeloid-cell classification. (C) Unpaired dot plots showing quantification of myeloid-cell subset frequencies by scRNA-seq (left) and flow cytometry (right). (D) Differential transcription factor activity across myeloid-cell subsets in healthy controls (HCs) and ST patients.

**Figure S8 Inferred transcriptional-state trajectory of myeloid-cell subsets.** (A) Pseudotime trajectory analysis of seven myeloid-cell subsets, colored by subset identity, pseudotime, and disease group. (B) Dynamic changes in gene expression and enriched biological processes along the myeloid-cell predicted differentiation trajectory.

**Figure S9 Functional enrichment of myeloid cells and plasma proteomic validation.** (A) Functional enrichment network analysis of myeloid-cell associated genes, highlighting pathways involved in inflammatory responses, innate immunity, antigen presentation, cytokine production, and immune regulation. Node color indicates myeloid-cell subset identity and node size reflects the number of genes contributing to each pathway. (B) Volcano plots showing differentially expressed plasma proteins between patients (n = 9) and healthy controls (n = 6). The y-axis represents -log_10_(*q* value) (left) and -log_10_(*p* value) (right). Proteins with *p* < 0.05 and *q* < 0.05 were labeled.
